# Costing of helicopter emergency services- a strategic simulation based on the example of a German rural region

**DOI:** 10.1186/s13561-020-00287-8

**Published:** 2020-10-08

**Authors:** Johann Röper, Markus Krohn, Steffen Fleßa, Karl-Christian Thies

**Affiliations:** 1 Chair of General Business Administration and Health Care Management, Faculty of Law and Economics, University of Greifswald, Friedrich-Loeffler-Straße 70, 17489 Greifswald, Germany; 2 Department of Anesthesiology and Intensive Care Medicine, University Medical Center Greifswald, Ferdinand-Sauerbruch-Straße, 17475 Greifswald, Germany

## Abstract

**Background:**

Helicopter emergency services (HEMS) are of increasing relevance for emergency medical services (EMS) of developed countries. Despite the known cost intensity of HEMS, there is only very limited knowledge of its cost dynamics and structures. This averts an efficient resource allocation of scarce EMS resources in an environment that is characterized by socio-political, medical and economic challenges. The objective of this study is the exemplary modeling of HEMS cost structures.

**Methods:**

We defined three scenarios with each five variations to illustrate different models of HEMS provision. Into these, we included varying availability times, technical features for off-shore or alpine rescue and differing numbers of operations. Cost data is based on a broad literature review and primary data from a German HEMS organization resulting in a cost function. We calculated average costs per primary missions and total costs, whilst differentiating between fixed, jump-fixed, variable and maintenance costs for every scenario variation. The costs were further used to evaluate the profitability of operations by executing a break-even analysis.

**Results:**

Average costs per HEMS operation decrease with increasing number of operations due to the digression of fixed costs. Depending on special equipment, availability times or other assumptions, total costs differ significantly with the different scenario variations. For the basic scenario (12 h of operations per day), the total costs per year of HEMS are 1,697,546.20 € and the unit costs are 763.41 € per primary mission at 1200 primary and 92 secondary operations. At an engine-runtime based revenue of 70 € per minute, global cost covering is possible after 728 missions (c.p.).

**Conclusions:**

Considering a revenue of 70 € per minute of engine run-time, HEMS can be operated at a profit for companies. However, the necessary remuneration represents a high financial effort for the societal cost bearers of helicopter emergency services. This leads to the question of the cost-benefit ratio of HEMS, which could be approached in further researches by using this model. The valuation of mission costs also opens a new view to the framework of HEMS disposition procedures and criteria. This cost analysis enhances the necessity of better planning of HEMS networks to use available resources efficiently in order to improve social welfare.

## Introduction

### Background

Helicopter Emergency Services (HEMS) are a substantial part of many national emergency medical service (EMS) systems. There is evidence that HEMS are beneficial to the most critical patients in the first place, although such patients are just a small fraction of the entire caseload of an average EMS system [1]. HEMS are characterized by its high resource intensity, which comprises scarce financial, technical or temporal resources or available manpower [2]. Therefore, the efficiency of HEMS has been questioned throughout, particularly in the wake of dwindling health care resources. In this field of tension, HEMS are faced with a constantly changing political, social, ethical, and medical environment.

Moreover, demographic and epidemiologic changes represent impending challenges. These appear particularly tangible in rural regions and will further enhance the importance of air supported emergency services. Consequently, the organization and delivery of rescue services must avoid the waste of resources in order to be able to use every available means for the best possible supply of emergency medical services. For this reason, the principle of economic efficiency must be applied, whereas its adherence requires theoretical and practical contributions from economic research.

In spite of the well-known cost-intensity of air rescue and contrary to the ongoing lively arguments about the location of HEMS bases, to the author’s knowledge there is only insufficient research knowledge from an economic perspective. Neither a formalization nor an analysis of the cost systematics of air rescue has been carried out yet in a scientifically satisfactory way [3]. The lack of scientific knowledge averts objective insights that could inform the political discussion and guide towards a more efficient allocation of resources. Ultimately, better macroeconomic assessments of the benefit of helicopter emergency medical services (HEMS), e.g. by comparison with medical outcomes, is not possible without a profound understanding of the dynamics of the operational cost.

### Aims

The aim of this study is to determine the full costs of an average air rescue operation under different scenarios, and thus make a fundamental contribution to an economic and business evaluation. The chosen scenarios take up on current public and political discussions with the aim to illustrate the dynamics and complexity of the German HEMS system. However, it is the intent of this study to generalize findings for wider, international use. The study’s underlying exemplary object of experience is the primary response helicopter system “Christoph 47”, which is based in Greifswald in the North East of Germany [4]. In order to deepen the understanding of HEMS operations, it is important to comprehend its prerequisites, the processes, the market and the social perspective. To achieve this, the general conditions of air rescue in Germany are described from an economic perspective.

In this regard, the provision of medical services is considered the existential justification for air supported emergency medical services. Medical services can generally be characterized by the uno-actu principle, which implies the unity of space, location and time of all partakers in the production of services. This principle is also valid in medical emergency situations, where its fulfilment is time critical [5]. Therefore, air medical services represent a process in the creation of health services, with the mission to provide professional medical help in out-of-hospital emergencies, with the number of provisions representing the cost driver, when highly utilized.

### EMS in Germany

In Germany, the emergency medical services are based on the principle, that the doctor comes to the patient, and not the patient to the doctor [6]. In contrast to many other English-speaking countries, the German EMS-system is historically based on a “stay-and-play” rather than a “scoop-and-run” field tactic [7]. Therefore, the German EMS maintains medics as medical support to the emergency physicians, but does not give them as far-ranging competences as known of the paramedic system [7, 8].. The German prerequisite holds for true the national HEMS, which first came to use in the 1970s to supplement ground EMS. Helicopters were usually deployed for primary missions, meaning the provision of the fastest patient transfer possible from the incident site to the best suited, nearest, hospital whilst being treated by a doctor [8].

After implementing the use of air transport to shorten the prehospital period in medical emergencies, the current German HEMS-System developed into an extensive network of over 80 helicopters in Germany serving more than 100,000 missions per year [9]. Previously mainly used for primary missions, secondary air transports of patients to tertiary care centers are experiencing a growing significance [10]. Some helicopters are specially equipped for critical care secondary transports, and can even accommodate heart-lung machines. Depending on geographical characteristics, the area of operations may pose challenges like rescues in hardly accessible places. In these cases, hoists or static ropes are carried. Both operations are usually primary missions required in mountainous or coastal regions [11].

Formally, most helicopters serve a strict area of application to supply means of primary or secondary transport. However, helicopters for primary transport in fact do secondary missions and vice versa, if only for smaller shares of the overall number of missions. In some cases, HEMS-stations are given the remit to serve both primary and secondary transport missions and are then called Dual-Use-Helicopters within the EMS. Consequentially, the formal division of the single-purpose use of secondary and primary helicopters is becoming increasingly indistinct [12, 13, 9].

The exemplary primary transport helicopter (PTH) in Greifswald serves a rural mission area of northeastern Germany with a low population density of less than 60 inhabitants per km^2^. This region is characterized by scarce infrastructure and geographical barriers, such as rivers, lagoons and islands. In its operating hours from 7 am to sunset, it provides help in over 1400 missions per year, which is within the common range of a highly utilized PTH in Germany [9]. In 2017, 90% of the overall missions were primary emergency deployments with a mean traveling distance of 29,11 km (18,09 miles), while secondary transports covered 47,23 km (29,34 miles) on average. The aircraft is an EC 145 (BK 117 C2) with 254 km/h (157,8 mph) cruise speed and does not carry extra rescue equipment, e.g. a hoist [4]. However, installing extra kit on the aircraft has been subject to various discussions. Since July 2019, the “Christoph 47” helicopter carries blood and defrosted fresh plasma reserves on board and thereby is the first German helicopter to do so [14]. Further, the extension of operating hours from sunrise to midnight is currently under discussion [15].

### Research regarding costs

To the best of the authors knowledge, there is only one study addressing a cost systematization of helicopter emergency medical services, by Fleßa et al., from 2016 [2]. It includes costs for personnel, fixed and variable costs. The study estimates costs of € 52,60 per airborne mission minute or € 1180 per average mission in a setting with 18 h of operations per day. However, Fleßa et al. do not take into account maintenance intervals. Neither are types of missions differentiated, such as by day or night, secondary or primary transports nor is miscellaneous equipment and its impact on costs considered.

Besides the study presented above, the German project PrimAir [16] explored the possibility of substituting ground EMS through HEMS in rural regions. In this context, a cost estimation for 24/7 air medical support was published, but without presenting a formal and systematic costing. The cost statement per year included day and night rescue services with a modern aircraft type H145, operated by 2 pilots in 1000 missions lasting an average 30 min. The study states full costs per year of 4,834,157 €, distinguishing between mission dependent and independent costs, the latter causing 25% of all costs. The PrimAir study further highlights the intensity of fixed costs of HEMS services. This information may be used for later benchmarking, but does not suffice to gratify this study’s aim.

Further research is compiled in a systematic literature review published in 2009 [3] by Taylor et al. concerning different economic evaluations of HEMS that are associated with benefits for patients. 15 articles that received a full review were classified in cost analysis, −minimization, −effectiveness and -benefit. Comparison of the estimated annual costs for HEMS show a 21-fold variation from 115,777 $ to 2,436,178 $. This leads the authors to conclude that a full economic evaluation is still overdue and vital to ultimately address a cost-effectiveness of HEMS.

### HEMS financing

The major source of remuneration for German HEMS-companies is linked to the number of operations and their duration, with valuation based on average missions or per flight minute. Prices are negotiated by insurance companies on behalf of the federal state as trustee [17, 18], there is no free pricing in the market. Further revenues are created through donations and sponsorships.

A general level of flight minute prices in the German HEMS system cannot be given, since they are determined state-specifically and vary considerably, despite similarities in operation frequencies, the equipment of the helicopters and the helicopter type. For example, the 12 HEMS-stations operated by the federal department of civil defense are compensated with 43.94 € per minute in flight [19]. Remuneration of these stations is independent from mission quantity, which varied in 2018 between 885 and 1607 total missions [20]. Further research predominantly shows reimbursements per flight minute of up to 80.10 € [21]. Other methods of reimbursement are based on missions and applicated with other German non-profit organizations. The exemplary price of an average primary mission is 2455.32 € and that of secondary missions is 4801.89 € [22]. When operating machines with special equipment, such as hoists or fixed rescue cables, even higher remunerations are to be expected.

Considering the significantly differing prices per flight minute and mission quantities, the question arises if a HEMS station can operate at a cost coverage or profit, and if it does, at what number of missions. Especially private non-profit organizations, that hold a large majority of the German HEMS market [23], may have less margin for operations under regular losses than the federal department for civil protection. Thus, answering the profitability question is also of socio-economic importance and could enhance efficiency of resource allocation in emergency medical systems.

## Methods

### Analysis

This study’s objective is to determine the unit cost of one primary mission of helicopter emergency medical services based on total costs. In compliance to the “Christoph 47’s” commission to provide medical support during the prehospital period, the relevant cost driving variable in this study is the number of primary missions during daytime.

A classic linear cost function consists of output independent fixed costs and variable costs, which are output dependent. Therefore, average cost developments are characterized by dispersion of fixed costs on a rising output quantity. Variable costs are constant and determine the marginal cost in linear functions. The decrease of average costs is ultimately limited by the constant variable cost [24].

Cost influencing parameters of helicopters exceed the classic structures of cost functions. Fixed and variable costs occur, as well as jump-fixed maintenance cost. Broken down into intervals, maintaining the aircraft’s airworthiness is not only influenced by the number of missions and therein the number of take-offs, but also by their duration. Within either maintenance interval the maintenance costs remain fixed and show the typical characteristics of fixed costs, but jump to a higher level when the next maintenance interval is triggered.

The scenarios and variations established in the following introduce different cost assumptions, that partly impact cost structures. To take these features into account, we derived the following cost function to calculate both total costs and unit costs of primary HEMS missions. The function is composed of variable costs per minute with running engines, jump-fixed maintenance, personnel and fixed costs.

Total costs are calculated as

$$ K=\mathrm{a}+\mathrm{p}+o+v+\sum \limits_{d=1}^4 trunc\left(\frac{\mathrm{r}+\left(1-\upalpha \right)\ast \mathrm{t}\ast \mathrm{x}+\upalpha \ast x\ast \mathrm{t}\ast \mathrm{m}+\mathrm{t}\ast \left(\mathrm{y}+\mathrm{z}+\mathrm{w}\right)}{u_d}\right)\ast {c}_d $$

$$ +\sum \limits_{d=1}^4 trunc\left(\frac{\mathrm{r}\ast \mathrm{s}+\left(1-\upalpha \right)\ast \mathrm{x}\ast {b}_x+\upalpha \ast {b}_x\ast \mathrm{x}\ast \mathrm{n}+\mathrm{y}\ast {b}_y+\mathrm{z}\ast {b}_z+\mathrm{w}\ast {b}_w}{e_d}\right)\ast {f}_d+ trunc\left(\frac{\alpha \ast x+r}{g}\right)\ast h $$

$$ +i\ast \left(x+y\right)+j\ast \left(z+w\right)+l\ast \left[\left(1-\alpha \right)\ast x\ast {b}_x+\mathrm{y}\ast {b}_y+\mathrm{z}\ast {b}_z+\mathrm{w}\ast {b}_w+\alpha \ast x\ast {b}_x\ast n\right] $$

Variable costs comprise the valued average use of medical equipment per mission type and fuel consumption for the duration of engine runtime. Maintenance cost are determined by two different jump-fixed cost categories that are triggered both by the number of starts and the runtime of the helicopter’s engines. When factoring in the transportation and use of special equipment aboard the helicopter, such as static ropes or rescue hoists, changing flight profiles with different take off frequencies and durations have to be considered. Furthermore, higher maintenance, personnel and other fixed costs have to be included, as well as the impact of flight times on variable costs.

The use of this special equipment does not represent a different type of mission. The described special operations have the aim to reduce prehospital times and can therefore be classified as primary missions that have different properties to the basic ones. The function for special equipment includes different primary mission durations and take-offs concerning the maintenance cost, extra fixed costs for better trained personnel and special equipment as well as changed fuel cost.

The calculation of the average costs per primary mission is based on the previously presented function for total costs as

$$ k=\frac{\mathrm{K}}{\mathrm{x}}=\frac{\frac{\mathrm{a}}{\upbeta +\upgamma}\ast \upbeta}{\mathrm{x}\ast {b}_x+\mathrm{y}\ast {b}_y}\ast {b}_x+\frac{\frac{\mathrm{p}}{\upbeta +\upgamma}\ast \upbeta \ast q}{\mathrm{x}\ast {b}_x}\ast {b}_x+\frac{o+v}{CEIL\left(\alpha \ast x\right)}\ast \alpha +l\ast \left(\left(1-\alpha \right)\ast {b}_x+\alpha \ast {b}_x\ast n\right)+i+\frac{trunc\left(\frac{\alpha \ast x+r}{g}\right)\ast h\ }{CEIL\left(\alpha \ast x\right)}\ast \alpha $$

$$ +\frac{\sum_{d=1}^4 trunc\ \left(\frac{\mathrm{r}+\left(1-\upalpha \right)\ast \mathrm{t}\ast \mathrm{x}+\upalpha \ast x\ast \mathrm{t}\ast \mathrm{m}+\mathrm{t}\ast \left(\mathrm{y}+\mathrm{z}+\mathrm{w}\right)}{u_d}\right)\ast {c}_d+{\sum}_{d=1}^4 trunc\left(\frac{\mathrm{r}\ast \mathrm{s}+\left(1-\upalpha \right)\ast \mathrm{x}\ast {b}_x+\upalpha \ast {b}_x\ast \mathrm{x}\ast \mathrm{n}+\mathrm{y}\ast {b}_y+\mathrm{z}\ast {b}_z+\mathrm{w}\ast {b}_w}{e_d}\right)\ast {f}_d}{\mathrm{r}\ast \mathrm{s}+\left(1-\upalpha \right)\ast \mathrm{x}\ast {b}_x+\upalpha \ast {b}_x\ast \mathrm{x}\ast \mathrm{n}+\mathrm{y}\ast {b}_y+\mathrm{z}\ast {b}_z+\mathrm{w}\ast {b}_w} $$

$$ \ast \frac{\mathrm{r}\ast \mathrm{s}+\left(1-\upalpha \right)\ast \mathrm{x}\ast {b}_x+\upalpha \ast {b}_x\ast \mathrm{x}\ast \mathrm{n}}{\mathrm{x}} $$

As EMS helicopters usually serve both primary and secondary transport missions, we established an allocation key to distribute fixed and personnel costs on either type of mission. The key is based on the area in km^2^ in which primary and secondary missions usually occur.

To determine under what circumstances either HEMS scenario turns profitable, the economic standard methodology of the break-even analysis is used. The break-even point shows the exact output level, at which revenue covers production costs and surpasses it whilst generating a profit. The calculation of the break-even point in this case is based on the overall duration of all missions measured in minutes, with a focus on primary missions. The overall mission duration is then multiplied with the fixed revenue per billable flight minute. Subtracting total costs from total revenues shows the profitability of HEMS operations given a certain number of missions.

$$ 0=P\ast \left(\left(1-\upalpha \right)\ast \mathrm{x}\ast {b}_x+\upalpha \ast {b}_x\ast \mathrm{x}\ast \mathrm{n}+\mathrm{y}\ast {b}_y+\mathrm{z}\ast {b}_z+\mathrm{w}\ast {b}_w\right)-K $$

The parameters used for the functions are:

**Table:** 

a	Fixed costs for standard operations in €	o	Additional fixed costs for special equipment in €
*b*_*x*_	Average billable duration of primary missions at daytime	p	Fixed personnel costs for standard operations in €
*b*_*y*_	Average billable duration of primary missions at nighttime	q	Share of personnel costs for daytime operations
*b*_*z*_	Average billable duration of secondary missions at daytime	r	Fixed number of yearly starts for special equipment training flights
*b*_*w*_	Average billable duration of secondary missions at nighttime	s	Average duration of training flights with special equipment
c	Cost for start-dependent maintenance interval in €	t	Number of starts per mission
d	Maintenance interval	u	Start-dependent maintenance interval
e	Flight-time dependent maintenance interval	v	Additional personnel fixed costs for special equipment operations in €
f	Cost for flight-time dependent maintenance interval in €	w	Number of secondary missions at nighttime
g	Usage dependent maintenance interval for rescue winch	x	Number of primary missions at daytime
h	Cost for usage dependent maintenance interval of the winch in €	y	Number of primary missions at nighttime
i	Value of average consumption of medical materials in € in primary missions	z	Number of secondary missions at daytime
j	Value of average consumption of medical materials in € in secondary missions	∝	Share of total primary missions with special equipment
k	Average costs of x in €	β	Area of operations for primary missions in km^2^
K	Total costs in €	γ	Area of operations for secondary missions in km^2^
l	Value of average fuel consumption per minute in €	P	Revenue per flight minute in €
m	Correction variable for number of starts per mission	trunc	Returns the integer and removes fractional numbers
n	Correction variable for billable mission duration	CEIL	Returns the least greater integer

### Scenarios

This cost analysis is based on three scenarios that are differentiating between HEMS operating hours. The scenarios further comprise four different variations that are in close relation to present public discussions and originate from the initial variation: high and low cost assumptions, the Dual-Use concept, winches and a static rope as extra rescue equipment.

**Table:** 

	Description	Variation
Scenario 1	12 operating hours from 7:00 am	a. Initial scenario b. Cost assumption: high c. Dual-Use services d. Extra equipment static rope e. Extra equipment winch
Scenario 2	16 operating hours from 7:00 am	
Scenario 3	24 operating hours	

Generally, the primary and secondary missions described above can be performed in daylight, in darkness or respectively at nighttime, when visual flight regulations (VFR) [25] apply. In this cost model, a differentiation between day- and nighttime operations is only being made for operations over 24 h per day. As for 12- and 16-h readiness, it is assumed that the (night) shift regulations do not apply to the crew despite late evening operating hours. The four mission types are included in the scenario analyses with an assumed number of annual deployments. These result from the historical deployment figures of the “Christoph 47” and the “Christoph Gießen” dual-use helicopter used as a supplement [9].

The construction costs of new HEMS bases in particular have already been criticized on various occasions, e.g. the German Federal Audit Office [17]. The initial scenario, in which low costs are assumed, is compared to an alternative “expensive” scenario in order to show the effect of the infrastructure costs on the full operational costs.

The mission figures included in the scenario analysis are as follows:

### Data

#### Personnel costs and other fixed costs

Table 2 gives a detailed explanation of the input data used and their allocation to cost categories for the determination of an average primary HEMS rescue mission. In this study, the fixed costs comprise the required ground infrastructure to run a HEMS station, aircraft dependent cost and personnel cost. As the regular mission field determines the requirements towards the machines, aircraft-dependent fixed costs are affected accordingly. While primary transport helicopters (PTH) serve a mission radius of about 50 to 70 km, determined by required EMS response times, secondary transport helicopters (STH) may fly several hundred kilometers to transfer patients between hospitals. Thus, deployed aircraft models vary in size, weight, engine performance, medical-technical installations or cabin volume, crew composition and therefore work space.

The initial scenario variation *I* corresponds to primary data of the German non-profit organization Johanniter Luftrettung (Air rescue division of Germany’s order of St. John’s) and to the exemplary object of experience. In this study’s basic scenario modeling, we assumed an older, but instrument flight capable, helicopter type BK 117 C2 with an operating life of 20 years. As the aircraft’s value is greatly dependent of its age, different assumptions concerning the helicopters acquisition cost and other equipment, e.g. for special rescue missions, seen as fixed costs, are taken into account in the different scenarios, as shown in Table 1.

**Table 1:** Scenario modeling

Scenario		1	2	3	1	2	3	1	2	3	1	2	3	1	2	3
Variation		***I*** Initial scenario			***II*** Cost assupmtion: High			***III*** Dual-Use services			***IV*** Extra equipment: static rope			***V*** Extra equipment: Rescue winch		
Number of missions	x	Variable			Variable			Variable			Variable			Variable		
	y	–	–	120	–	–	120	–	–	120	–	–	120	–	–	120
	z	92	92	92	92	92	92	400	400	400	92	92	92	92	92	92
	w	–	–	140	–	–	140	–	–	140	–	–	140	–	–	140

In particular, the acquisition costs of the rescue equipment, the construction and upkeep of the air rescue station infrastructure can be identified from the outset as fixed cost drivers. Besides obligatory costs for building maintenance, insurances, cleaning of premises and laundry or office equipment, another factor is the depreciation on facilities. In this full cost analysis, it is assumed that infrastructure is the HEMS operator’s property, so that the possibility of a leasing cost can be neglected.

Table 2 shows other fixed costs concerning the staff needed to run a HEMS station for 12-, 16- or 24 h a day. In general, German and international HEMS operate with at least one pilot, one emergency physician and one medic, called HEMS-Technician (TC), who receives special training to support the pilot when not otherwise occupied. During night flights in 24/7 operations, as subject of scenario 3, German flight regulations generally call for a second pilot [25]. However, new concepts enable medics to aid pilots in instrument flight rule conditions, when operating over 16 h per day, like illustrated in scenario 2 [26]. The HEMS-TC-NVIS is then trained in the use of night vision imaging systems to assist the pilot during night time operations. Personnel costs include the total cost for the employer.

**Table 2:** Input Data

Scenario			1					2					3				
**Variation**			**I**	**II**	**III**	**IV**	**V**	**I**	**II**	**III**	**IV**	**V**	**I**	**II**	**III**	**IV**	**V**
**x**	**Number of primary missions at daytime**		**variable**														
**y**	**Number of primary missions at nighttime**		**0**	**0**	**0**	**0**	**0**	**0**	**0**	**0**	**0**	**0**	**120**	**120**	**120**	**120**	**120**
**z**	**Number of secondary missions at daytime**		**92**	**92**	**400**	**92**	**92**	**92**	**92**	**400**	**92**	**92**	**92**	**92**	**400**	**92**	**92**
**w**	**Number of secondary missions at nighttime**		**0**	**0**	**0**	**0**	**0**	**0**	**0**	**0**	**0**	**0**	**140**	**140**	**140**	**140**	**140**
**bx** **by**	**Average billable duration of primary missions at day- and nighttime**		**20 min**														
**bz** **bw**	**Average billable duration of secondary missions at day- and nighttime**		**45 min**														
**Yearly fixed costs in €**																	
**Helicopter**	**Purchase cost**	Helicopter type BK 117 C2 with glas cockpit, life cycle 20 years	155.250 €	450.000 €	155.250 €	155.250 €	155.250 €	155.250 €	450.000 €	155.250 €	155.250 €	155.250 €	155.250 €	450.000 €	155.250 €	155.250 €	155.250 €
		2 night vision goggles (NVG)	0 €	0 €	0 €	0 €	0 €	22.000 €	22.000 €	22.000 €	22.000 €	22.000 €	22.000 €	22.000 €	22.000 €	22.000 €	22.000 €
	**Medical devices and ambulance kit**^**a**^	Life cycle 5 years	50.000 €	50.000 €	50.000 €	50.000 €	50.000 €	50.000 €	50.000 €	50.000 €	50.000 €	50.000 €	50.000 €	50.000 €	50.000 €	50.000 €	50.000 €
	**Consumption of other medical material per year**		2.000 €	2.000 €	2.000 €	2.000 €	2.000 €	2.000 €	2.000 €	2.000 €	2.000 €	2.000 €	2.000 €	2.000 €	2.000 €	2.000 €	2.000 €
**Station cost**	**Facility equipment**	Kitchen, bathrooms, relaxation and recreation areas, office, IT	15.000 €	30.000 €	15.000 €	15.000 €	15.000 €	15.000 €	30.000 €	15.000 €	15.000 €	15.000 €	15.000 €	30.000 €	15.000 €	15.000 €	15.000 €
	**Facility upkeep**	Energy costs	6.000 €	4.000 €	6.000 €	6.000 €	6.000 €	6.000 €	6.000 €	6.000 €	6.000 €	6.000 €	6.000 €	4.000 €	6.000 €	6.000 €	6.000 €
		Cleaning of premises and clothing	7.200 €	10.000 €	7.200 €	7.200 €	7.200 €	7.200 €	7.200 €	7.200 €	7.200 €	7.200 €	7.200 €	10.000 €	7.200 €	7.200 €	7.200 €
		Building insurance	3.000 €	3.000 €	3.000 €	3.000 €	3.000 €	3.000 €	3.000 €	3.000 €	3.000 €	3.000 €	3.000 €	3.000 €	3.000 €	3.000 €	3.000 €
		Maintenance	6.000 €	6.000 €	6.000 €	6.000 €	6.000 €	6.000 €	6.000 €	6.000 €	6.000 €	6.000 €	6.000 €	6.000 €	6.000 €	6.000 €	6.000 €
		Construction (life cycle 50 years)	40.000 €	60.000 €	40.000 €	40.000 €	40.000 €	40.000 €	40.000 €	40.000 €	40.000 €	40.000 €	40.000 €	60.000 €	40.000 €	40.000 €	40.000 €
	**Vehicle**	Life cycle 6 years	10.000 €	20.000 €	10.000 €	10.000 €	10.000 €	10.000 €	10.000 €	10.000 €	10.000 €	10.000 €	10.000 €	20.000 €	10.000 €	10.000 €	10.000 €
	**Outstanding receivables**		15.000 €	15.000 €	15.000 €	15.000 €	15.000 €	15.000 €	15.000 €	15.000 €	15.000 €	15.000 €	15.000 €	15.000 €	15.000 €	15.000 €	15.000 €
	**Insurances**		7.000 €	7.000 €	7.000 €	7.000 €	7.000 €	7.000 €	7.000 €	7.000 €	7.000 €	7.000 €	7.000 €	7.000 €	7.000 €	7.000 €	7.000 €
**a**	**Sum of fixed costs**		**316.450 €**	**657.000 €**	**316.450 €**	**316.450 €**	**316.450 €**	**338.450 €**	**648.200 €**	**338.450 €**	**338.450 €**	**338.450 €**	**338.450 €**	**679.000 €**	**338.450 €**	**338.450 €**	**338.450 €**
Technical equipment	Winch^b^		0 €	0 €	0 €	0 €	94.980 €	0 €	0 €	0 €	0 €	94.980 €	0 €	0 €	0 €	0 €	94.980 €
	Fixed cable^c^		0 €	0 €	0 €	39.980 €	0 €	0 €	0 €	0 €	39.980 €	0 €	0 €	0 €	0 €	39.980 €	0 €
**v**	**Sum of special fixed costs**		**0 €**	**0 €**	**0 €**	**39.980 €**	**94.980 €**	**0 €**	**0 €**	**0 €**	**39.980 €**	**94.980 €**	**0 €**	**0 €**	**0 €**	**39.980 €**	**94.980 €**
**Yearly personnel costs in €**																	
**Scenario variation**			**1.I**	**1.II**	**1.III**	**1.IV**	**1.V**	**2.I**	**2.II**	**2.III**	**2.IV**	**2.V**	**3.I**	**3.II**	**3.III**	**3.IV**	**3.V**
Personnel	**Pilots**		360.000 €	420.000 €	360.000 €	360.000 €	360.000 €	480.000 €	560.000 €	480.000 €	480.000 €	480.000 €	720.000 €	840.000 €	720.000 €	720.000 €	720.000 €
	**Physicians**		200.000 €	240.000 €	200.000 €	200.000 €	200.000 €	200.000 €	240.000 €	200.000 €	200.000 €	200.000 €	310.000 €	372.000 €	310.000 €	310.000 €	310.000 €
	**HEMS-TC**		130.000 €	140.000 €	130.000 €	130.000 €	130.000 €	130.000 €	140.000 €	130.000 €	130.000 €	130.000 €	195.000 €	217.000 €	195.000 €	195.000 €	195.000 €
	**Training to HEMS-TC-NVIS**		0 €	0 €	0 €	0 €	0 €	10.000 €	10.000 €	10.000 €	10.000 €	10.000 €	0 €	0 €	0 €	0 €	0 €
	**Administration**		15.000 €	20.000 €	15.000 €	15.000 €	15.000 €	15.000 €	20.000 €	15.000 €	15.000 €	15.000 €	15.000 €	15.000 €	15.000 €	15.000 €	15.000 €
	**Airworthiness examination**		14.000 €	14.000 €	14.000 €	14.000 €	14.000 €	14.000 €	14.000 €	14.000 €	14.000 €	14.000 €	14.000 €	14.000 €	14.000 €	14.000 €	14.000 €
	**General training cost**		5.000 €	8.000 €	5.000 €	5.000 €	5.000 €	20.000 €	20.000 €	20.000 €	20.000 €	20.000 €	32.000 €	32.000 €	32.000 €	32.000 €	32.000 €
**p**	**Sum of personnel costs of scenario variation in €**		**724.000 €**	**842.000 €**	**724.000 €**	**734.000 €**	**769.000 €**	**869.000 €**	**1.004.000 €**	**869.000 €**	**879.000 €**	**914.000 €**	**1.286.000 €**	**1.490.000 €**	**1.286.000 €**	**1.306.000 €**	**1.331.000 €**
**q**	**Share of personnel costs at daytime**		**1**	**1**	**1**	**1**	**1**	**1**	**1**	**1**	**1**	**1**	**4/7**	**4/7**	**4/7**	**5/9**	**4/7**
Training	**Training to HEMS-TC-HHO**		0 €	0 €	0 €	0 €	10.000 €	0 €	0 €	0 €	0 €	10.000 €	0 €	0 €	0 €	0 €	10.000 €
	**Training for special equipment winch**		0 €	0 €	0 €	0 €	35.000 €	0 €	0 €	0 €	0 €	35.000 €	0 €	0 €	0 €	0 €	35.000 €
	**Training for special equipment fixed cable**		0 €	0 €	0 €	10.000 €	0 €	0 €	0 €	0 €	10.000 €	0 €	0 €	0 €	0 €	10.000 €	0 €
**o**	**additional personnel cost for special equipment**		**0 €**	**0 €**	**0 €**	**10.000 €**	**45.000 €**	**0 €**	**0 €**	**0 €**	**10.000 €**	**45.000 €**	**0 €**	**0 €**	**0 €**	**10.000 €**	**45.000 €**
**Maintenance costs and intervals**																	
Start dependent maintenance intervals	Interval **d**	**u**_**d**_ **- Starts**	**c**_**d**_														
	1	200	4.200 €	4.200 €	4.200 €	4.200 €	4.200 €	4.200 €	4.200 €	4.200 €	4.200 €	4.200 €	4.200 €	4.200 €	4.200 €	4.200 €	4.200 €
	2	400	8.000 €	9.000 €	8.000 €	8.000 €	8.000 €	8.000 €	9.000 €	8.000 €	8.000 €	8.000 €	8.000 €	9.000 €	8.000 €	8.000 €	8.000 €
	3	1200	50.000 €	55.000 €	50.000 €	50.000 €	50.000 €	50.000 €	55.000 €	50.000 €	50.000 €	50.000 €	50.000 €	55.000 €	50.000 €	50.000 €	50.000 €
	4	2400	100.000 €	110.000 €	100.000 €	100.000 €	100.000 €	100.000 €	110.000 €	100.000 €	100.000 €	100.000 €	100.000 €	110.000 €	100.000 €	100.000 €	100.000 €
Flight time dependent maintenance intervals	Interval **d**	**e**_**d**_ **- Hours**	**f**_**d**_														
	1	3000	1.000 €	1.000 €	1.000 €	1.000 €	1.000 €	1.000 €	1.000 €	1.000 €	1.000 €	1.000 €	1.000 €	1.000 €	1.000 €	1.000 €	1.000 €
	2	6000	3.000 €	3.000 €	3.000 €	3.000 €	3.000 €	3.000 €	3.000 €	3.000 €	3.000 €	3.000 €	3.000 €	3.000 €	3.000 €	3.000 €	3.000 €
	3	18,000	14.000 €	15.000 €	14.000 €	14.000 €	14.000 €	14.000 €	15.000 €	14.000 €	14.000 €	14.000 €	14.000 €	15.000 €	14.000 €	14.000 €	14.000 €
	4	36,000	40.000 €	45.000 €	40.000 €	40.000 €	40.000 €	40.000 €	45.000 €	40.000 €	40.000 €	40.000 €	40.000 €	45.000 €	40.000 €	40.000 €	40.000 €
Winch maintenance intervals	Interval **d**	**g**_**d**_ **- Usage**	**h**_**d**_														
	1	1	0 €	0 €	0 €	0 €	100 €	0 €	0 €	0 €	0 €	100 €	0 €	0 €	0 €	0 €	100 €
	2	20	0 €	0 €	0 €	0 €	3.000 €	0 €	0 €	0 €	0 €	3.000 €	0 €	0 €	0 €	0 €	3.000 €
**Other cost parameters**																	
**r**	starts per training with special equipment		0	0	0	300	100	0	0	0	300	100	0	0	0	300	100
**s**	training duration in minutes per start with special equipment		0	0	0	20	60	0	0	0	20	60	0	0	0	20	60
***∝***	share of special equipment operations of x		0	0	0	0,05	0,1	0	0	0	0,05	0,1	0	0	0	0,05	0,1
**l**	Rated average fuel consupmtion per minute (traveling speed 254 km/h and 318 l/h)		5,83 €														
**i**	Rated average consumption of medical materials per primary operations		120 €														
**j**	Rated average consumption of medical materials per secondary operations		170 €														
**t**	starts per mission		2														
**m**	correction variable for special equipment mission starts		1	1	1	1,5	0,5	1	1	1	1,5	0,5	1	1	1	1,5	0,5
**n**	correction variable for special equipment mission duration		1	1	1	2	2	1	1	1	2	2	1	1	1	2	2
***β***	area for primary operations		11,550 km^2^														
***γ***	area for secondary operations		23,000 km^2^														

^a^Includes: Hamilton respirator, Corpuls C3 ECG-Defibrillator unit, 2 Medumat Transport Intensive respirators, 4 Braun Compact Perfusors, Akkuvac exhaust pumps, Sonosite ultrasound, emergency pack children and adults, kits for ventilation, traumatology, vacuum mattress, immobilization

^b^Composure: 300.000 € acquisition cost, life cycle 5 years, 100 training flights per year á 60 min, fuel consumption cf. variable costs

^c^Composure: 5000 € acquisition cost, life cycle 1 year, 100 training flights per year á 60 min, fuel consumption cf. variable costs

When carrying special missions equipment as illustrated in scenario-varieties *IV* and *V* (compare Table 1), such as medical devices, winches or fixed ropes for use in situations with problematic patient accessibility, more or better trained personnel supplement the standard crew. Higher training levels, e.g. helicopter hoist operators (HEMS-TC-HHO), are consequentially regarded with higher personnel costs.

#### Maintenance costs

In this model, the usage-dependent maintenance costs for ensuring the operational safety of the rescue equipment are driven by the engine runtime and by the number of take-offs. These are reflected in four maintenance cycles of varying extent. This study generally assumes two starts for both primary and secondary missions on the grounds of historical flight data [9].

When including rescue winches in scenario-variety *V*, a proportion of 10% of all primary missions is assumed. Using a winch presumably allows nonstop flights but causes twice the average primary mission flight time, as the helicopter does not have to land to board the patient, but hovers above the emergency location. Further, extra maintenance intervals for the winch are taken into account, due to its rather complex technique.

In case of static cables as in scenario-variety *IV*, the proportion of primary missions is 5%. This assumption is based on a lower range of application compared to the rescue winch. When executing static cable rescues, the cable is installed after a reconnaissance flight, the patient then picked up and brought to a stopover landing place and taken on board. Fixed cable missions therefore require three starts and presumably twice the average primary mission flight time. Due to its technical simplicity, extra maintenance intervals for fixed cables are not regarded, and checks are presumably absorbed in regular training flights.

#### Variable costs

The variable costs of air rescue depend on the number of missions and their duration in terms of the engine runtime, which in this study is considered in minutes. This includes kerosene consumption, which is valued at the net price of Jet A-1 fuel [4]. Assumed average fuel consumption per minute is derived from the data sheet of the BK 117 C2 used in Greifswald [4]. The medical material required for operation is also taken into account as the average consumption value per mission.

## Results

### Average costs analysis

**Fig. 1:**
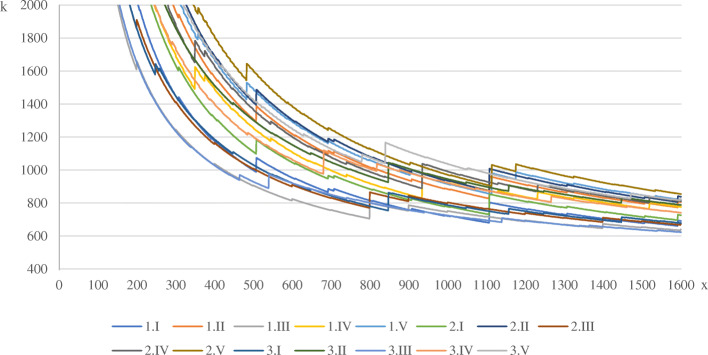
Development of primary mission average costs

The development of the average cost per primary HEMS-mission is shown in Fig. 1. Evidently, an increase in mission number results in a strong digression of average cost for every scenario. Further, the decrease of average primary mission cost is visibly broken by the triggering of maintenance intervals, which represent jump-fixed cost and lead to a sudden increase of the average costs on multiple occasions. The cost components for select mission numbers as presented in the specific scenarios will subsequently be listed.

#### Scenario 1

Table 3 shows the average fixed, personnel, variable, average and full costs per primary rescue mission at daytime for the mission numbers as used in the scenario *1* modeling. Further, the total costs for all operations based on the modeling are presented. When comparing each scenario variation at 1200 missions, highest cost appear when operating a winch with 973.70 € (c.p.). The lowest average costs arise when operating a dual-use helicopter as presented in scenario *1.III* with 695.43 € per average primary mission. Considering the underlying real life scenario *1.I* with 1500 primary missions per year, the average cost per primary rescue mission c.p. is 678.79 €. About 34.86% of these are distributed on variable and 30% on maintenance cost, whilst personnel costs cause 23.77%.

**Table 3:** Cost overview scenario 1

	x	z	Avg. fixed cost	Avg. Personnel cost	Avg. maintenance cost	Avg. variable cost	Avg. cost for primary missions	Total cost for primary missions	Total cost
**Scenario 1.I**	900	92	117.54 €	268.92 €	135.32 €	236.60 €	758.39 €	682,549.10 €	1,438,366.20 €
	1200	92	88.16 €	201.69 €	236.96 €	236.60 €	763.41 €	916,090.16 €	1,692,946.20 €
	1500	92	70.53 €	161.35 €	210.31 €	236.60 €	678.79 €	1018,186.21 €	1,789,526.20 €
**Scenario 1.II**	900	92	244.04 €	312.75 €	144.35 €	236.60 €	937.75 €	843,971.58 €	1,906,916.20 €
	1200	92	183.03 €	234.57 €	256.15 €	236.60 €	910.34 €	1,092,410.28 €	2,178,496.20 €
	1500	92	146.42 €	187.65 €	226.71 €	236.60 €	797.39 €	1,196,083.18 €	2,276,076.20 €
**Scenario 1.III**	900	400	58.77 €	268.92 €	222.56 €	236.60 €	786.85 €	708,166.16 €	1,689,166.20 €
	1200	400	50.38 €	201.69 €	206.76 €	236.60 €	695.43 €	834,516.77 €	1,793,746.20 €
	1500	400	44.08 €	161.35 €	212.42 €	236.60 €	654.45 €	981,674.74 €	1,940,326.20 €
**Scenario 1.IV**	900	92	173.08 €	272.64 €	163.10 €	242.43 €	851.25 €	766,123.85 €	1,524,993.20 €
	1200	92	129.81 €	204.48 €	261.03 €	242.43 €	837.75 €	1,005,295.45 €	1,781,322.20 €
	1500	92	103.85 €	163.58 €	260.81 €	242.43 €	770.67 €	1,155,998.83 €	1,933,651.20 €
**Scenario 1.V**	900	92	273.08 €	285.64 €	165.88 €	248.26 €	972.86 €	875,572.68 €	1,654,740.20 €
	1200	92	204.81 €	21,423 €	306.41 €	248.26 €	973.70 €	1,168,443.58 €	1,971,118.20 €
	1500	92	163.85 €	171,38 €	265.33 €	248.26 €	848.82 €	1,273,235.98 €	2,071,496.20 €

The highest total cost when considering 1200 missions occur under the high-cost scenario *1.II* with 2,178,496.20 €, followed by scenario *1.V* with about 2 million €. Initial scenario *1.I* shows the lowest costs of 1,697,546.20 € given the mission quantity.

#### Scenario 2

Table 4 shows the average fixed, personnel, variable, average and full costs per primary rescue mission at daytime for 16 daily operating hours, as well as the full costs for all operations based on the modeling shown in Table 1. Similar to scenario *1*, when comparing each scenario variation at 1200 missions, the highest average costs appear when operating a winch with 1020.23 € (c.p.). The lowest average cost arise when operating a dual-use helicopter as presented in scenario *2.III* with 739.33 € per average primary mission. Considering the expanded underlying real life scenario *2.I* with 1500 primary missions per year, the average cost per primary rescue mission c.p. is 809.93 €. Of these, about 29% are distributed on either variable, maintenance or personnel costs.

**Table 4:** Cost overview scenario 2

	x	z	Avg. fixed cost	Avg. Personnel cost	Avg. maintenance cost	Avg. variable cost	Avg. cost for primary missions	Total cost for primary missions	Total cost
**Scenario 2.I**	900	92	125.71 €	322.78 €	135.32 €	236.60 €	820.42 €	738,376.88 €	1,605,366.20 €
	1200	92	94.29 €	242.09 €	236.96 €	236.60 €	809.93 €	971,917.94 €	1,859,946.20 €
	1500	92	75.43 €	193.67 €	210.31 €	236.60 €	716.01 €	1,074,013.99 €	1,956,526.20 €
**Scenario 2.II**	900	92	240.77 €	372.93 €	144.35 €	236.60 €	994.65 €	895,186.05 €	2,060,116.20 €
	1200	92	180.58 €	279.70 €	256.15 €	236.60 €	953.02 €	1,143,624.75 €	2,331,696.20 €
	1500	92	144.46 €	223.76 €	226.71 €	236.60 €	831.53 €	1,247,297.65 €	2,429,276.20 €
**Scenario 2.III**	900	400	62.86 €	322.78 €	222.56 €	236.60 €	844.80 €	760,316.66 €	1,856,166.20 €
	1200	400	53.88 €	242.09 €	206.76 €	236.60 €	739.33 €	887,192.61 €	1,960,746.20 €
	1500	400	47.14 €	193.67 €	212.42 €	236.60 €	689.83 €	1034,744.56 €	2,107,326.20 €
**Scenario 2.IV**	900	92	181.25 €	326.50 €	163.10 €	242.43 €	913.28 €	821,951.63 €	1,691,993.20 €
	1200	92	135.94 €	244.87 €	261.03 €	242.43 €	884.27 €	1,061,123.24 €	1,948,322.20 €
	1500	92	108.75 €	195.90 €	260.81 €	242.43 €	807.88 €	1,211,826.62 €	2,100,651.20 €
**Scenario 2.V**	900	92	281.25 €	339.50 €	165.88 €	248.26 €	1034.89 €	931,400.47 €	1,821,740.20 €
	1200	92	210.94 €	254.62 €	306.41 €	248.26 €	1020.23 €	1,224,271.36 €	2138.118.20 €
	1500	92	168.75 €	203.70 €	265.33 €	24.26 €	886.04 €	1,329,063.76 €	2,238,496.20 €

The highest total cost when considering 1200 missions occur under the high-cost scenario *2.II* with 2,331,696.20 €, followed by scenario *1.V* with 2138,118.20 €. Given the selected mission count, by comparison the initial scenario *2.I* shows the least costs of 1,859,946.20 €.

#### Scenario 3

Table 5 shows the average fixed, personnel, variable, average and full costs per primary rescue mission at daytime in 24-h operations, as well as the full costs for all operations based on the modeling presented above. The selected mission numbers and associated costs are excerpts from Fig. 1. When comparing each scenario variation at 1200 missions, the highest cost occur when operating a winch with 930.81 € c.p. The lowest average cost arise when operating a dual-use helicopter as presented in scenario *3.III* with 691.62 € per average primary mission. Considering the scenario *3.I* at 1500 primary missions per year, the average cost per primary rescue mission c.p. is 701.50 €. Of these, about 33% are distributed on each variable deployment and maintenance cost, whilst personnel costs cause 23%.

**Table 5:** Cost overview scenario 3

	x	y	z	w	Avg. fixed cost	Avg. Personnel cost	Avg. maintenance cost	Avg. variable cost	Avg. cost for primary missions	Total cost for primary missions	Total cost
**Scenario 3.I**	900	120	92	140	110.92 €	268.92 €	218.81 €	236.60 €	835.26 €	751,730.14 €	2,291,887.20 €
	1200	120	92	140	85.71 €	201.69 €	226.38 €	236.60 €	750.39 €	900,470.53 €	2,442,467.20 €
	1500	120	92	140	69.84 €	161.35 €	233.71 €	236.60 €	701.50 €	1,052,254.28 €	2,597,047.20 €
**Scenario 3.II**	900	120	92	140	222.54 €	312.75 €	236.32 €	236.60 €	1.,008.21 €	907,387.74 €	2,863,437.20 €
	1200	120	92	140	171.96 €	234.57 €	244.84 €	236.60 €	887.97 €	1,065,563.20 €	3,021,017.20 €
	1500	120	92	140	140.12 €	187.65 €	252.85 €	236.60 €	817.22 €	1,225,825.19 €	3,182,597.20 €
**Scenario 3.III**	900	120	550	140	62.86 €	268.92 €	188.81 €	236.60 €	757.20 €	681,476.32 €	2,376,487.20 €
	1200	120	550	140	53.88 €	201.69 €	199.45 €	236.60 €	691.62 €	829,942.37 €	2,531,067.20 €
	1500	120	550	140	47.14 €	161.35 €	192.31 €	236.60 €	637.41 €	956,111.95 €	2,641,647.20 €
**Scenario 3.IV**	900	120	92	140	166.46 €	272.64 €	302.62 €	242.43 €	984.14 €	885,730.17 €	2,442,514.20 €
	1200	120	92	140	127.36 €	204.48 €	258.82 €	242.43 €	833.09 €	999,711.38 €	2,540,843.20 €
	1500	120	92	140	103.16 €	163.58 €	259.24 €	242.43 €	768.41 €	1,152,617.82 €	2,697,172.20 €
**Scenario 3.V**	900	120	92	140	266.46 €	285.64 €	311.75 €	248.26 €	1112.11 €	1,000,898.51 €	2,562,261.20 €
	1200	120	92	140	202.36 €	214.23 €	265.95 €	248.26 €	930.81 €	1,116,969.00 €	2,663,639.20 €
	1500	120	92	140	163.16 €	171.38 €	265.08 €	248.26 €	847.89 €	1,271,830.16 €	2,822,017.20 €

The highest total cost when considering 1200 missions occur under the high-cost scenario *3.II* with 3,021,017.20 €, followed by scenarios *1.V* with 2,663,639.20 €. Initial scenario *3.I* shows the least costs of 2,442,467.20 €, compared to the other varieties.

### Break even analysis

**Fig. 2:**
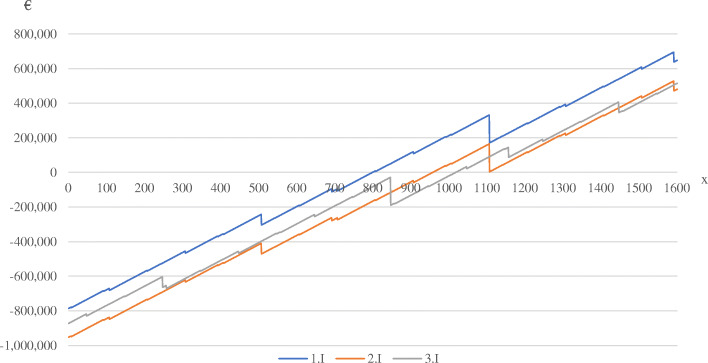
Break even analysis of standard scenario

Figure 2 shows the results of a break-even analysis for average primary HEMS missions, following the methodology presented above. It includes the total cost and total revenue of all operations as defined in the respective scenario. Underlying the revenue is a price of 70 € per mission minute with running engines. The presentation is restricted to the three basic scenario variations in order to point out the cost-revenue dynamics whilst obtaining a clear overview.

Figure 2 displays, that the break-even point of real-life scenario *1.I* is only attained after 800 primary flights, which are needed to balance uncovered costs, including the recognized secondary missions. After these missions, HEMS-operations remain profitable and unchallenged by possible maintenance costs. The presumption of 16 h of operations, as depicted in scenario *2.I*, turns profitable after 959 primary missions, all other things being equal. The total break-even point of scenario *2.I* is sustainable, even though the 1108th primary mission, under the presented assumptions, triggers a maintenance interval which significantly reduces former profits. The HEMS-operations over 24 h per day, as shown in scenario *3.I,* are the last to break even at 1020 missions. A first approach to breaking even at 847 missions c.p. fails, due to the entering of a maintenance interval.

## Discussion

### Cost analysis

The results of the helicopter emergency services cost simulation shown above represent a first comprehensive approach to fully depict the cost structures of air supported medical services. As displayed in the basic scenario 1a, the average cost for the concerning mission type ranges from 758 € at 900 missions to 678 € at 1500 missions. These results show considerably lower costs per mission than Fleßa et al. (2016) have calculated with 1180 €, although the approach to the modeling is certainly somewhat different to the one chosen in this work.

Total costs presented in the PrimAir study of 2016 for a scenario of 1000 missions per year with missions at day- and nighttime were calculated with 4.834.157 €, with a proportion of 25% being dependent on the number of missions. The corresponding approach of this analysis, represented in scenario *3.I*, indicates significantly lower total costs of 2.562.261,20 € at 900 primary missions. Furthermore, following the presented cost dynamics, 50,36% (sum of maintenance and variable costs) of the total costs are mission dependent. These strongly varying cost estimates should be further addressed and verified in future research.

The effect of high fixed costs is displayed by variations *I* and *II* in all scenarios and shows a significant impact on overall costs. Fixed costs are therefore a cost driver and should be kept low to ensure cost efficient HEMS-operations. Regarding the German helicopter emergency services, this is not always the case, as has frequently been criticized, most prominently by the German federal audit office [17].

As shown above, jump-fixed maintenance cost have a cost driving influence and may cause uncertainty, if and when the mission count triggers a new cost interval. As maintenance costs are immanent to HEMS operations and cannot be avoided, fleet expansion, standardization and occupancy optimization of maintenance facilities could help HEMS organizations to lower maintenance expenses. This could also substantiate the rationale of the investment in a fleet renewal. In fact, fleet expansion and standardization can be observed in many international HEMS organizations such as the swiss Rega, the German DRF Luftrettung (German Air Rescue) or the ADAC Luftrettung (Air Rescue of the General German Automobile Club). This justifies the assumption of scenario variation b, which contrasts the influence of higher maintenance costs to a lower cost assumption in the other variations.

HEMS-operators are faced with a make-or-buy decision concerning training costs, which is a driver of personnel costs. The importance of this decision will be heightened, when regarding future expansions of operating hours into nighttime, which might be imminent especially in rural regions. In Germany, e.g. the ADAC Luftrettung and DRF Luftrettung keep their own training academies, and may therefore be in a strategic position to set industry standards and gain advantages in service quality over competitors.

### Availability times

Most German HEMS-stations with a focus on primary missions operate only during daylight, and nighttime operations are usually secondary patient transfers within hospitals. Still, a feasible concept for night time emergency operations could be a huge opportunity to ensure better emergency medical services and higher aircraft utilisation. Regarding rural regions in the northern hemisphere with only few daylight hours during winter, this holds even more true. Excerpting the basic scenario variation *1.I*, this model calculates total costs of 1,789,526.20 € for 1500 primary and 92 secondary missions of the underlying helicopter model. An extension of operating hours from 12 to 16, whilst keeping the mission count constant, leads to a total cost increase of 8.5% to 1,956,526.20 €. Day and night operations as depicted in scenario *3.I* lead to total costs of 2,597,047.20 €, which implies an increase of 31.09% over basic scenario *1.I*, but also to an increase and further diversification of overall missions.

These findings lead to the question, why operating hours in Germany seem to be so restrained. Firstly, the duplication of HEMS availability from 12 on average to 24 h of operations implies significantly better emergency medical cover, especially in rural areas, where airborne EMS is of importance in fulfilling the legally required EMS response times (27). Secondly, as shown above, an increase of operating hours entails an under proportional cost increase. Therefore, in a medical sense, welfare could be heightened by expanded helicopter emergency medical service hours and justified by a favorable cost-benefit relation. It can be stated, that expanded operating hours lead to a higher cost efficiency concerning the relation between the provision of funds and the availability of resources.

### Break-even analysis

The break-even analysis that complements this cost analysis shows, that at a given number of missions in a period, helicopter supported medical services can be operated at a profit. The socio-economic question is therefore, whether public health services should be operated profitably and if so, how profitably should they be? As mentioned before, private non-profit organizations may have less margin for losses than those that are publicly held. Considering the lively competition between HEMS operators, which leads to a striving for cost reduction and performance improvements in the sense of economic efficiency, profits and periodical losses are system immanent. Cost coverage is thus a necessary precondition of private companies to fulfill the economic principle of prudence and to remain operational.

As shown in Fig. 2, the basic scenario variation *I* can be operated at a profit for every given time frame of operations. A remuneration of € 70 per flight minute seems to be a realistic price in the upper half regarding the German HEMS system. Likewise realistic is the highest break-even point by scenario *3.I* at 1020 missions, a quantity often exceeded by far by German HEMS aircrafts [9]. Given the presented presumptions, this simulation model therefore calculates a fairly large profit, that could be gained by certain German HEMS bases with high occupancy. For HEMS organizations that operate several stations, these profits might be needed so subsidize less utilized ones, for instance in rural areas with low population density or regions that are overlapped by neighboring posts. These would point to inefficiencies of HEMS networks in general.

However, the operation of a non-cost covering organizational unit seems questionable from the economic viewpoint of a HEMS non-profit organization and should be subject to further research. A possible explanation for the upkeep of stations with under-average profitability could be, amongst others, positive effects through higher utilization of maintenance facilities, cost savings in insurance policies, strengthening of the brand name or public awareness. Furthermore, HEMS organizations have no direct influence on a post’s mission quantity, as dispatch is done by the EMS control centers. Extreme mission quantities, utilization-based remuneration and thus economic profits may therefore be indicative of systemic, inefficient resource allocation. In particular, if profits are generated with medical emergency services, that are out of range of public interest.

Besides the profit-determinant number of missions in a certain period, a high occupancy of HEMS stations increases the danger of emergency duplicities, in which a helicopter is bound at one incident, although airborne medical support would be more important at a parallel one. To improve output efficiency of HEMS systems, the profile of HEMS missions should urgently be assessed for every station. A possible approach could be the analysis of NACA-Scores, with the aim to determine, whether helicopter based services should only be dispatched to the most severe situations by creating a mission profile with regard to individual stations. Results can be valued and opposed to the model that has been presented in this study.

## Conclusion

This full cost scenario analysis is based on an emergency medical service helicopter as an object of experience, which is mainly used for primary rescue operations. Our aim was to abstract cost structures and dynamics to gain a representative economic view over HEMS operations. This was approached by defining different scenario variations counting 15 in total. These comprised different cost assumptions concerning the helicopter’s equipment, its personnel, cost implications of longer times of operations or different cost of infrastructure. Cost data was obtained by approaching a number of air rescue providers in Germany with responsibility for more than 80 helicopters in total. Most data was provided by a German HEMS organization being responsible for 5 air rescue helicopters, operating mainly in the field of Dual-Use and secondary transports. The scenarios were designed to be widely transferable to other HEMS stations or systems, with the scenario variations taking into account possible discrepancies of HEMS stations or data accuracy.

This study shows that the cost of higher availability of a rescue helicopter, e.g. during night time, does not increase proportionally to the extended time frame. Furthermore, the average cost of a mission decreases significantly with increasing usage. At 1400 primary missions, which is common for primary rescue helicopters in Germany to be reached within 1 year, economies of scale disperse the fixed costs of HEMS to a degree, where average mission costs converge visibly (Fig. 1). This leads to the question, why primary rescue helicopters are not available 24/7 in Germany. From an economic and societal perspective, the usual availability of air supported medical services during daylight points to an inefficient resource allocation, as patients don’t have access to medical aid at all times. This study shows, that under the given conjectures, the cost-benefit ratio of extended operational hours is favorable.

Based on a revenue per billable minute of operations of 70 €, this study also shows the possible profitability of HEMS. The break-even point however depends on the cost assumptions of the respective scenario.

## Limitations

A major challenge was to collect and structure inputs that would be used to define different HEMS-scenarios. With little available studies that present cost data, we tried to partner with the four big HEMS organizations in Germany, the DRF Luftrettung, ADAC Luftrettung, Bundesamt für Bevölkerungs- und Katastrophenschutz (German Federal Office for Civil Protection and Disaster Protection) and the Johanniter Luftrettung. Unfortunately, only the Johanniter Luftrettung were willing to support this study in several meetings, although without presenting official documents. All primary data were protocolled during these meetings and now represent the HEMS cost input structure as realistic as possible.

Presumptions made in the different scenario variations are based on the initial scenario *1.I*, therefore the presented results may give a good impression of the costs that arise when operating air supported EMS, but cannot claim to perfectly mirror the exemplary object of experience. As it was not possible to empirically evaluate the cost systematics developed in this study, we have to leave this for further research. A comparison to already existing benchmarks has been discussed, however their inconsistence prevents a sound judgement of this studies results. The lifting of information barriers by all stakeholders would improve future research quality and open this widely unworked field in health care management.

## Data Availability

The datasets generated and analyzed in this study are available in Table 2 “Input Data” repository. Further information is available on request.

## Abbreviations

EMS: Emergency Medical Services
HEMS: Helicopter Emergency Medical Services
HEMS-TC: HEMS-Technician
HEMS-TC-HHO: HEMS-TC-Helicopter-Hoist-Operator
PTH: Primary Transport Helicopter
STH: Secondary Transport Helicopter
VFR: Visual Flight Regulations
